# Home-based HIV counseling and testing: Client experiences and perceptions in Eastern Uganda

**DOI:** 10.1186/1471-2458-12-966

**Published:** 2012-11-12

**Authors:** David Kyaddondo, Rhoda K Wanyenze, John Kinsman, Anita Hardon

**Affiliations:** 1 Department of Social Work/Child Health and Development Centre, Makerere University, Kampala, Uganda; 2 Department of Disease Control and Environmental Health, Makerere University School of Public Health, Kampala, Uganda; 3 Department of Public Health and Clinical Medicine, Umeå Centre for Global Health Research, Epidemiology and Global Health Unit, Umeå University, 901 85, Umeå, Sweden; 4 Centre for Social Science and Global Health, University of Amsterdam, Amsterdam, Netherlands

**Keywords:** Home-based HIV counseling and testing, Africa

## Abstract

**Background:**

Though prevention and treatment depend on individuals knowing their HIV status, the uptake of testing remains low in Sub-Saharan Africa. One initiative to encourage HIV testing involves delivering services at home. However, doubts have been cast about the ability of Home-Based HIV Counseling and Testing (HBHCT) to adhere to ethical practices including consent, confidentiality, and access to HIV care post-test. This study explored client experiences in relation these ethical issues.

**Methods:**

We conducted 395 individual interviews in Kumi district, Uganda, where teams providing HBHCT had visited 6–12 months prior to the interviews. Semi-structured questionnaires elicited information on clients’ experiences, from initial community mobilization up to receipt of results and access to HIV services post-test.

**Results:**

We found that 95% of our respondents had ever tested (average for Uganda was 38%). Among those who were approached by HBHCT providers, 98% were informed of their right to decline HIV testing. Most respondents were counseled individually, but 69% of the married/cohabiting were counseled as couples. The majority of respondents (94%) were satisfied with the information given to them and the interaction with the HBHCT providers. Most respondents considered their own homes as more private than health facilities. Twelve respondents reported that they tested positive, 11 were referred for follow-up care, seven actually went for care, and only 5 knew their CD4 counts. All HIV infected individuals who were married or cohabiting had disclosed their status to their partners.

**Conclusion:**

These findings show a very high uptake of HIV testing and satisfaction with HBHCT, a large proportion of married respondents tested as couples, and high disclosure rates. HBHCT can play a major role in expanding access to testing and overcoming disclosure challenges. However, access to HIV services post-test may require attention.

## Background

HIV testing and counseling is the gateway to prevention, care and treatment since these interventions depend on individuals seeking HIV testing and knowing their HIV status [1]. Additionally, studies have suggested that early initiation of HIV treatment may have important prevention benefits [2-4]. Despite the importance of HIV Counseling and Testing (HCT), its uptake remains low, particularly in developing countries. HIV testing in fact remains one of the greatest challenges to current HIV/AIDS policy [5,6]. Globally, over 60% of HIV-infected individuals remain unaware of their sero-status [7]. In Sub-Saharan Africa, it is estimated that 80% are unaware of their status and nearly 90% are unaware of their partner’s status [8].

Global concern over the gulf between the needs and the reality has led to urgent calls for increased access to HCT services [9]. Recent years have witnessed new initiatives to increase access, including incorporating HCT into routine healthcare and providing HCT within people’s homes (Home-Based HIV Counseling and Testing or HBHCT). National and international policies have also been revised to incorporate provider-initiated testing and counseling (PITC) [10]. In Uganda options for getting tested are no longer restricted to stand-alone Voluntary Counseling and Testing (VCT) facilities since HCT services were integrated within broader healthcare and HBHCT was adopted in 2005 [11]. The number of people ever tested for HIV duly increased from 23% in 2006; to 38% in 2008, and 57% in 2010/11 to 57% in 2010/11 [12-15].

Within HBHCT, providers reach out to the community, providing counseling and testing within clients’ homes. In Uganda, HBHCT comes in three different forms. First, some research and surveillance programs include HBHCT as part of their research [14,15]. The second approach is family-based or targeted ‘index client’ HCT, mainly used by HIV treatment programs, where the homes of HIV-infected patients registered into care are identified, visited and household members tested for HIV. The third approach, the one used in the area where this study was conducted, is door-to-door HBHCT, where mobile teams of counselors and testers mobilize entire communities and provide HCT door-to-door [11].

HBHCT has been lauded for making testing convenient. It has been commended for its success in increasing uptake, with acceptance rates of over 90% [16-19]. The high uptake of HBHCT is associated with the elimination of costs for clients traveling to VCT centers and the removal of stigma associated with going to such centers [16,17,20]. Menzies and colleagues noted the importance of door-to-door HBHCT for reaching many first-time testers, including couples and children, and for the early identification of HIV infection. They also noted that HBHCT, at US$ 8.29 per client, is comparatively cheaper than hospital-based HCT (US$ 11.68) and stand-alone VCT (US$ 19.26) [17]. Other benefits of HBHCT include the opportunity to reach the entire household with HIV interventions [21].

While there are many advantages to HBHCT, our limited understanding of its processes necessitates further study [22,23]. More specifically, concerns surround: 1) privacy – due to the general lack of counseling space at home [18]; 2) confidentiality – whether health workers can keep clients’ results from being revealed to household members and neighbors; 3) consent – whether testing at home is really voluntary and whether the right to refuse is not down-played by providers [24]; and 4) whether clients diagnosed with HIV infection are referred and successfully linked to appropriate medical care. Some observers remain cautious about the potential for negative effects in HBHCT, including stigma, discrimination and violence that may come with disclosure of HIV-positive status [25-27]. Similarly, there are concerns about consent and the tension between safeguarding individual rights and protecting public health [9,28,29].

This paper examines the experiences of HBHCT clients in Kumi district in eastern Uganda. We asked clients about: 1) the process of mobilization; 2) counseling, consent, privacy and confidentiality; 3) disclosure and 4) referral to care.

## Methods

This study was conducted as part of the Multi-country African study on Testing and Counseling for HIV (MATCH), designed to compare modes of testing for HIV across four countries: Burkina Faso, Kenya, Malawi and Uganda. The study was conducted in 2008–2009 and included a main survey of clients and providers at healthcare facilities as well as smaller surveys of people involved in home-based testing in Kenya and Uganda, and testing campaigns in Burkina Faso and Malawi. In 2007 and 2008, HBHCT was implemented in selected districts in Kenya and Uganda.

Kumi district (2,821 square kilometers) is located in eastern Uganda and has an estimated population of 370,800 (projected from the 2002 census). HIV prevalence in eastern Uganda is estimated at 5.3% (MoH and ORC Macro, 2006), while Kumi district authorities have estimated district prevalence to be 3.6%. Kumi has three hospitals (two public and one private) and 27 health centres. A number of these facilities provide HIV-related services, for example Prevention of Mother to Child Transmission (PMTCT). Kumi is the second district in Uganda to provide district-wide door-to-door HBHCT, after Bushenyi [30]. The program was implemented in 2007 by district health services with support from the President’s Emergency Plan for AIDS Relief (PEPFAR), through the US Centers for Disease Control and prevention (CDC). HBHCT has been implemented in more than 10 other districts in Uganda, including Bushenyi, Tororo and Kalangala, among others [31].

The district health team trained 29 teams of providers, each with a counselor, a laboratory technician, and mobilizers. The 29 teams moved simultaneously from house to house in the district’s 16 sub-counties, providing HCT services free of charge, using rapid test kits (Determine, Uni-Gold and STAT-PAK) and immediately disclosing results. The teams mobilized a specific number of homes each day, commensurate to their personnel capacity. This meant that they were able to allocate considerable time to conduct comprehensive individual counseling – something that would not be possible in public facilities where providers have additional duties and no control over the number of clients coming to seek services.

Prior to the HBHCT team’s visit, community mobilizers ensured that all households were informed of its coming. Mobilizers mainly consisted of local community leaders, though other community resource persons were also trained for this role. Outreach activities were organized in public places such as churches and mosques while the district health team held talk shows on the local FM radio stations to mobilize communities for HBHCT. People who were married or cohabiting were encouraged to test as couples rather than individuals. Over 95% of people who were approached in Kumi district agreed to be tested with a reported prevalence of 4% [22].

Between April and June 2008, we conducted 395 individual interviews in Kumi district. Kumi was selected because it had recently completed the HBHCT exercise (within six months of this study). This was intended to minimize recall bias.

We adopted the WHO 30 x 7 cluster sampling strategy (27). Previously used in studies to estimate coverage levels in the Expanded Program on Immunization (EPI), it employs a simplified cluster sampling method based on the random selection of 210 respondents in 30 clusters (7 respondents per cluster).

In this study, 32 clusters were selected from four (two rural and two urban) sub-counties. For each stratum (rural and urban sub-county), 16 villages (clusters) were randomly selected to provide a total of 32 clusters. Seven households were selected in each village (cluster). In the rural areas where households are scattered, adjacent households were visited. In the urban and peri-urban areas where households are close to each other, we selected every fifth household to avoid selecting all respondents from the same vicinity. In each household, we interviewed up to two eligible respondents. All adults (male and female) in a household (18 years and older) were eligible for participation. The minimum number of respondents would be 224 (32x7) if only one respondent was interviewed per household. However, the total number of respondents was 395 because we targeted to interview two respondents in every household (except those households where only one person was available at the time of the interview) [32]. Using the rotary method, respondents were randomly selected from those adults present in their homes at the time of the interviews. Overall, 65% of our respondents were women. Men were probably under-represented in the population that we sampled, because they work more often outside the community (the interviews were done during the day).

We used semi-structured questionnaires to elicit information on the HBHCT processes, from mobilization to counseling, testing, receipt of results and post-test experiences (disclosure and access to services). All respondents provided written informed consent either by signature or thumb print (for those who were unable to write). Participants were asked about the information that they received before testing; whether they consented to or declined to be tested; their perceptions of consent and counseling processes pre- and post-test; disclosure of HIV status and the outcomes of disclosure; and referral to care for those who were found to be HIV-positive.

As our goal was to examine the HBHCT processes, we excluded those respondents (114) who reported testing before the HBHCT program started in 2007. Clients’ experiences were analyzed following the trajectory of HBHCT: from the point of community mobilization to counseling before the test, the consent procedure, post-test counseling and receipt of results, disclosure dynamics and for those found to be HIV-positive, referral to care. We used largely descriptive statistics to document respondents’ experiences. The analysis was done by gender to check for gender variations in experiences (p < 0.05). We also analyzed the open-ended questions that were embedded in our questionnaires using ‘cloud analysis’ software to identify common themes, which describe client experiences and perceptions across the HBHCT trajectory. Quantitative data were analyzed using the Stata statistical package (2010).

The study was approved the ethics committee of the Amsterdam Medical Center, the Makerere University Child Health and Development Centre, and the Uganda National Council for Science and Technology.

## Results

Out of the 395 individuals interviewed, 376 (95%) had ever tested for HIV. Among the 19 who had never tested, six said they had been approached for testing through HBHCT but declined to test. The majority of those who had never tested (12 out of 19) were men. Out of the 376 respondents who had ever tested for HIV, 281 (75%) had done so through HBHCT in or after 2007. Of this latter group, 12 (4%) reported being HIV-positive while 262 (96%) stated that they were not infected; seven declined to declare their HIV status. The experiences of testing at home described below concern the 281 respondents who tested through HBHCT in or after 2007.

Most HBHCT clients (65%) were married or cohabiting and had little formal education. Cultivation was the leading occupation (51%), with only 10% in professional employment and 18% in trade(see Table 1).

**Table 1:** Social demographic characteristics of respondents

**Characteristic**	HBHCT users*		**HBHCT non-users**	
	**Total N= 281**	**Total %**	**Total N=19**	**Total %**
**Age (1 missing value)t**				
Under 20	26	9.3%	2	10.5%
20-24	48	17.1%	2	10.5%
25-29	48	17.1%	5	26.3%
30-34	50	17.8%	4	21.1%
35-39	27	9.6%	3	15.8%
40-44	31	11.0%	0	0%
Over 45	50	17.8%	3	15.8%
Missing value	1	0,4%		
**Gender**				
Female	182	64.8%	7	36.8%
Male	99	35.2%	12	63.2%
**Religion**				
Catholic	148	52.7%	13	68.4%
Muslim	9	3.2%	1	5.3%
Pentecostal	30	10.7%	1	5.3%
Protestant/Church of Uganda	94	33.5%	4	21.1%
**Education**				
No formal education/incomplete primary	163	58%	11	57.9%
Primary	35	12,5%	0	0%
Secondary/vocational	69	2,6%	7	36,8%
Post secondary or higher	9	3.2%	0	0%
Missing value	5	1.8%	1	5.3%
**Marital status**				
Never married	60	21.4%	8	42.1%
Married or cohabiting	183	65.1%	9	47.4%
Divorced/separated	15	5.3%	1	5.3%
Widowed	23	8.2%	1	5.3%
Type of work**				
Agriculture	142	50.5%	12	63.2%
Homemaker/housewife	59	21.0%	2	10.5%
Commerce/trade	51	18.1%	1	5.3%
Student	24	8.5%	2	10.5%
Professional/employed	27	9.6%	1	5.3%
Skilled/semi-skilled	20	7.1%	2	10.5%
Unskilled	18	6.4%	2	10.5%
Unemployed	5	1.8%	2	10.5%
Fishing	1	0.4%	-	-
Other	12	4.3%	-	-
**HIV status**				
Negative	262	95.6%		
Positive	12	4.4%		
Unknown			19	100%

*HBHCT users are those who tested through HBHCT.

P** Some respondents were engaged in more than one type of work.

In Table 2 the experiences of HBHCT are presented by gender, and in Table 3 the referral experiences are given for the 12 respondents who tested HIV positive.

**Table 2:** Experience of HBHCT clients: mobilization, consent, confidentiality and counseling

**Experience**	**Result total**	**Results by gender**	
	**N=281 (%)**	**Female N=182 (%)**	**Male N=99 (%)**
**Mobilization**			
Informed about the visit of the HBHCT team	218(77.6)	147(80.8)	71(71.7)
HBHCT providers:			
1. Came alone	209 (74,4)	136 (74.7)	73 (73.7)
2. Came with local council leaders	57 (20.2)	40(21.9)	17 (17.2)
3. Came with others	15(5.5)	6(3.3)	9(9.1)
**Consent process**			
Provider asked if client agreed to testing	271 (96.4)	176 (96.7)	95 (96.0)
Respondent considers it important to be asked if he/she agrees			
1. Very important	245(87.2)	156(85.7)	89(89.9)
2. Somewhat important	16(5.7)	14(7.7)	2(2.0)
3. Not important	20(7.1)	12(6.6)	8(8.1)
Provider explained option to decline	264 (94.0)	170 (93.4)	94^1^ (94.9)
Other in the household also offered a test	171 (60,9)	109 (59,9)	62 (62.6)
Response of other household members offered a test			
1. All accepted	116 (67.8)	73 (67.0)	43 (69.4)
2. Some accepted, some refused	45 (26.3)	28 (25.7)	17 (27.4)
3. All refused	8 (4.7)	7 (6.4)	1 (1.6)
4. Don’t know	2 (1.2)	1 (0.9)	1 (1.6)
Discussed test with other household members before consenting	90 (52,9)	57 (52,8)	33 (53,2)
Influenced by what other household members said or did	36 (40.0)	24 (42.1)	12^2^(36.4)
**Counseling**			
Received counseling before the test	254 (90.4)	163 (89.6)	91 (91.9)
Tested alone/individual	188 (74.0)	129 (79.1)	59 (64.8)
Where pre-test counseling was done:			
1. Inside house	120 (48.0)	73 (45.9)	47 (51.6)
2. Outside	129 (51.6)	86 (54.1)	43 (47.3)
Provider explained how test works, pre-test	207 (81.5)	128 (78.5)	79^3^ (86.8)
Provider explained meaning of positive and negative results, pre-test	236 (84.0)	153 (84.1)	83^4^ (83.8)
**Provider explained meaning of test result, post-test**	**272 (98.2)**	**174 (97.2)**	**98 (100.0)**
Provider explained window period post-test	221 (87.7)	140 (86.4)	81 (90)
Provider gave opportunity to ask questions, post-test	234 (93.2)	148 (92.5)	86 (94.5)
Provider gave advice on prevention of HIV, post-test	264 (95.3)	169 (94.4)	95 (96.9)
**Confidentiality**			
Client told that results will remain confidential	246 (97.6)	159 (98.1)	87 (96.7)
How the client valued confidentiality			
1. Very important	218 (78.7)	141 (78.8)	77 (78.6)
2. Somewhat important	17 (6.1)	13 (7.3)	4 (4.1)
3. Not important	41 (14.8)	24 (13.4)	17 (17.3)
Client felt results were kept confidential			
1. Yes	196 (70.8)	126 (70.4)	70 (71.4)
2. No	9 (3.2)	7 (3.9)	2 (2.0)
3. Don’t know	71 (25.6)	45 (25.1)	26 (26.5)
**Disclosure**			
Provider suggested sharing results with someone	149 (53.8)	99 (55.3)	50 (51.0)
Other people in the household shared results with respondent	125 (45.1)	76^5^ (42.5)	49 (50.0)
Asked by household member to share results	79 (28.5)	48^6^ (26.8)	31 (31.6)
Neighbors asked if respondent took an HIV test	136 (49.1)	86^7^ (48.0)	50 (51.0)
Neighbors asked to share HIV test results	76 (27.4)	49^8^ (27.4)	27 (27.6)
Generally keep results confidential	186 (66.2)	126^9^ (69.2)	60^10^ (60.6)
Has disclosed to someone	219 (77.9)	144^11^ (79.1)	75^12^ (75.8)
People disclosed to (*multiple responses*)			
Spouse/partner	135 (61.6)	90 (62.5)	45 (60.0)
Parents	68 (31.1)	46 (31.9)	22 (29.3)
Children	42 (19.2)	27 (18.8)	15 (20.0)
Siblings	32 (14.6)	22 (15.3)	10 (13.3)
Other relatives	57 (26.0)	30 (20.8)	27 (36.0)
Friends*	99 (45.2)	55 (38.2)	44 (58.7)
Client has discussed HIV status with someone in household*	155 (56.0)	92^13^ (51.4)	63 (64.3)
**Satisfaction**			
Received sufficient information	259 (93.5)	165 (92.2)	94 (95.9)
Meeting with provider was helpful	262 (94.6)	167 (93.3)	95 (96.9)
**Overall treatment by provider**			
Very well	168 (60.6)	106 (59.2)	62 (63.3)
Well	52 (18.8)	34 (19.0)	18 (18.4)
Okay	53 (19.1)	35 (19.6)	18 (18.4)
Badly	4 (1.4)	4 (2.2)	0 (0.0)

*significant p.

1. 1 missing value.

2. 1 missing value.

3. 1 missing value.

4. 1 missing value.

5. 3 missing values.

6. 2 missing values.

7. 1 missing value.

8. 1 missing value.

9. 3 missing values.

10. 2 missing values.

11. 3 missing values.

12. 2 missing values.

13. 1 missing value.

**Table 3:** Referral to care and support for those tested positive

**Referral service**	**Number (%) N=12**
Told medication was needed	11 (91.7)
Given further medical/blood tests	9 (75.0)
Does client know CD4 count	5 (55.6)
Referred to medical care	8 (66.7)
Has obtained medical care	7 (87.5)
Advised to join HIV & AIDS support group	11 (91.7)
Referred to a PHA support group	11 (91.7)
Has joined support group	8 (66.7)
Received assistance (e.g. food)	3 (25.0)

### Mobilization for HBHCT

The majority (78%) of respondents received prior information about the visit of the HBHCT team; mobilizers went beforehand to households alerting people about the planned HBHCT exercise; 20% of the respondents reported that local leaders accompanied the testing team.

### Dynamics of Consent

Consent procedures are one of the concerns raised about HBHCT. Can people opt out when the testing team visits their homes? 271 out of 281 HBHCT clients (96%) said the providers had asked if they agreed to the HIV test; 94% of the respondents stated that HCT providers had informed them of their right to decline testing. The opportunity to opt-out was highly valued by respondents; when asked how important it was for them to agree to the testing, 87% said it was very important.

The majority of respondents (61%) reported that more than one person in their household was offered HBHCT services. About half of respondents (53%) said they discussed HIV testing with one or more household members before consenting to the test. Some people initially declined but later accepted after other household members agreed to be tested. Patterns of influence varied. Respondents reported:

“Actually for me, my parents and siblings had a discussion on HIV testing and so my decision was influenced.”

“Being the mother of the home I influenced my son.”

“Daddy sat with me and talked to me and encouraged me to test for HIV.”

There were also instances of adult children influencing their parents to take the HIV test. Some clients discussed the HIV test with household members to seek encouragement and approval: *“It is after talking to my auntie that I become strong and tested.”*

But even after such discussions, some respondents felt that the decision to take the test was ultimately their own: *“Though we had discussed it, each one finally had to make a personal decision.”“Though I had discussed this with my husband, I had to make a personal decision at the end of the day.”“Though I had discussed this with my wife, at the end of the day I had to make a decision on my own.”* Others felt that their discussion with the HBHCT service provider had the greatest impact: *“My decision was actually influenced by what the health providers talked to me about and not by my father.”*

### Pre- and post-test counseling

Most respondents (90%) stated that they were counseled before the test, and 94% of clients felt that the information they received from providers was sufficient and useful.

Of those who received pre-test counseling, the majority (74%) received one-on-one counseling. However, we found high rates of couple counseling among the married/co-habiting respondents (N=49): 69% were counseled and tested with their partner. Regarding the physical location where the counseling took place, 52% reported that it was done outside the house, while 48% reported that they were counseled inside the house.

The quality of counseling was high (see Table 2); 95% of the respondents reported that they received information on prevention in post-test counseling. The majority (93%) stated that they were allowed to ask questions during the post-test counseling session. Respondents were satisfied with the interactions. Most respondents (94%) said the information they received was sufficient; 95% thought the meeting with the provider was helpful.

### Confidentiality

Almost all respondents (98%) stated that they had been assured by the HBHCT team that their results would remain confidential. When asked about the importance of confidentiality, 78% said it was very important, while 6% said it was somewhat important, and 15% said it was not important.

One respondent described HBHCT as *“a wonderful practice that allows a lot of privacy.”* Most respondents reported that HBHCT allowed confidentiality and freedom of discussion between clients and providers: *“I think it is good because when people are reached individually in their own homes they can open up easily.”*

Some respondents stated that HCT in the privacy of their own homes allows for greater confidentiality than testing in health facilities, where there are many other people: *“It’s very good [testing at home] because some people fear going to hospital, the reason being that if they are found positive other people will know about their status.”*

Regarding the protection of test results by providers, the majority of respondents (71%) felt that their results were protected, including 10 out of the 12 who reported testing HIV-positive; only 3% reported that they felt their results were not kept confidential.

### Disclosure dynamics

Overall, 49% of HBHCT clients reported that neighbors had asked them if they had been tested, and 28% had been asked to share their HIV test results; 29% reported that their household members had asked them to share their results. There were no significant differences between men and women’s experiences in this regard. An almost equal proportion of women (79%) and men (76%) reported that they had disclosed to someone; of those who had disclosed 63% of women and 60% of men did so to their spouse/partners. A larger proportion of men than women reported that they had discussed their HIV status with *other* members of the household (64% versus 51%, p=0.058). Similarly, a larger proportion of men (59%) than women (38%) had disclosed to their friends (p=0.006). Slightly more women than men reported that they generally keep their status confidential (69% versus 61%).

Among the 12 respondents who reported to be HIV-positive, all had disclosed to someone. All HIV positive individuals who were married or cohabiting had disclosed to their partners (one via their mother-in-law). Participants acknowledged disclosure and asking a partner to test as a difficult process and gave varied experiences. One HIV infected young man, for example, said he had spoken to his wife about having a test: “*I told her that we should go and get tested so that if we are HIV positive we start treatment…I was sure I did not have HIV because I tried being faithful to my partner.”* He says he disclosed to his wife because *“she had a right to know my status”.* At first she was distressed and wished that they hadn’t gone for testing, but *“now we have accepted our status”*, he says.

One HIV infected woman reported that she was alone in the house during home-based testing. When she found out that she was positive she disclosed to her husband, as *“there is no way I was going to hide this”*. Describing how he reacted, she says he was rude and did not want anything to do with testing or taking drugs. He wanted to break-up. But she said: *“lately he has changed and now he is supportive”*.

Another HIV infected woman narrated how her mother in-law asked her what her test result was. *“I told her what the counselor had told me”*. She said that her co-wife subsequently asked whether it was true. She decided to tell her, and was relieved that her co-wife *“did not react badly”*, but instead supported her and encouraged her to start treatment.

Half of those who tested HIV-positive did not think it was important.

### Referral to care

Eleven out of 12 clients who tested HIV positive said they needed medication but only eight said that they had been referred to medical care by the HBHCT providers and seven had received care. When probed about their health status, five said they knew their CD4 counts. Eleven HIV-positive respondents were counseled to join support groups and 8 of them had done so.

While most respondents valued the privacy of their homes for testing, several of the HIV positive respondents felt that testing at a health facility had advantages over testing at home: *“When you are at the health centre you easily find out about other health problems rather than [testing for] HIV alone.”* One HIV positive respondent remained skeptical about HIV testing at home, noting that: *“It’s a good idea but I am not sure if their results are as genuine as the hospital results.”*

In general, our respondents stated that HBHCT had enabled those who were infected to initiate treatment after learning of their HIV status as one of them commented: *“It is good, even my sick auntie who could not go to hospital was tested and found positive; now she is on medication.”“It is very good, even people who were bed-ridden got tested and now they are on treatment; they are even better than they were before they knew they were positive.”*

## Discussion

This study revealed high levels of uptake of HIV testing and counseling and overall satisfaction with HBHCT. The percentage of respondents who had ever tested was much higher than the national average of 38% at the time [13]. All individuals who tested received their results immediately, facilitated by the use of rapid test kits. Similar results have been reported by other HBHCT studies andprograms [16-18,30,33], suggesting that if expanded, HBHCT could increase uptake of HIV testing and reach many first-time testers.

The quality of services and the attitude of healthcare providers are often cited as limiting factors in the use of HIV counseling and testing services [31,34]. In our study, both male and female clients reported largely positive experiences with HBHCT: the information they received during pre- and post-test counseling, the consent procedure, and general handling by providers. Most respondents were counseled before the test and felt that the information they received from providers was sufficient. Allowing clients to ask questions was highly valued.

We found high adherence to consent requirements on the part of the HBHCT providers, in particular explanation of the opt-out option. Nearly all (94%) respondents (both male and female) acknowledged being given the chance to opt out of the test; and they valued this. The Kumi HBHCT teams were able to allocate considerable time to conduct comprehensive individual counseling since they controlled the number of households and individuals served on each day.

Contrary to our expectation that privacy within homes would be problematic due to limited space and the presence of other family members, clients considered their own homes as more private than healthcare facilities. Most respondents were satisfied with the privacy offered by HBHCT. Other studies have described the involvement of families in seeking private counseling space within and outside the home [20]. The participation of clients in identifying private spaces within the home was experienced as empowering, in contrast to facilities where users have no say over the setting of testing and counseling.

While most clients were satisfied with the level of privacy, more than a quarter cast doubt on whether their test results would be safeguarded after providers left their homes. In this study, no direct question was asked about breach of confidentiality by the provider. Nonetheless, in order to build trust and confidence, HBHCT clients should be informed about the practical issues of confidentiality beyond the test process and what happens to records when providers leave their homes.

It is widely assumed that individuals do not want others to know that they have tested for HIV [35]. In the context of HBHCT, anonymity may be impossible. Family members and neighbors knew about the visiting HBHCT team and tried to find out whether others had taken the test. Almost half of the respondents were asked by other community members if they had taken the HIV test. Nevertheless, we found that anonymity was not a major concern among respondents as there was no stigma attached to testing: most household and community members were in the same boat. Those who declined to test were the minority. This is unlike testing in a voluntary testing and counseling facility where lone individuals who go for testing prefer to hide their identity [36]. HBHCT opened up space within the home to talk about HIV and testing. To some extent, interactions and discussions among partners and other family members influenced individual decisions to test at home, though others stated, that testing was a personal responsibility. The dynamics of these discussions varied between respondents; patterns of influence often did not conform to the traditional hierarchy of many African families (e.g. parents over children, husbands over wives). For example, we found sons and daughters to have encouraged their parents to take the test.

Prior mobilization allows potential clients to think about, discuss and make a decision before the team arrives. This is similar to VCT where an individual can take time to consider whether or not to be tested. Individuals who do not wish to be tested could, for example, leave home before the arrival of the HBHCT team. The involvement of local leaders in the mobilization process can influence individual decisions to take the test; their involvement encouraged trust in the program and community cooperation. Although not a focus of this study, the widespread mobilization and discussions around HIV within the homes and community may change the norms around HIV testing and impact on HIV stigma, discrimination and access to services.

Disclosure levels were found to be high: 78% of HBHCT clients had disclosed their HIV test results to someone, often to more than one person. However, the disclosure was selective as reported in other studies [37,38]. Men were found to disclose significantly more to others in the household and to friends than women. This is an interesting finding, as other studies suggest that men are more secretive about their status than women. It is important to note here that 96% of our informants were negative. For those who test negative, disclosing is perhaps not a big deal. Disclosure problems are more likely to occur for those who test positive. However, some previous studies have demonstrated no increases in domestic violence or other negative social outcomes after HBHCT [39]. Our findings show that all of the HIV positive respondents had disclosed their results to someone. Many clients tested (69%) were counseled with their partners, suggesting that home-based testing is a good way to promote couple-counseling. Couple counseling and testing may also explain the high disclosure rates to sexual partners, in comparison to other studies [30,40].

The findings from this study may have some limitations: 1) Recall bias (some respondents had tested more than a year prior to the interview); 2) This study gathered information on only individuals who were present at home at the time of the interview, which may exclude the views of those who were out (at work); 3) The study generally relied on respondent accounts which may be subject to bias due to social desirability. However, in real life, it is users’ perceptions that trigger many health actions including care, and it is thus important to explore the client perspectives.

The number of HIV infected individuals was too small to exhaustively explore linkage to and access to HIV care and treatment as well as outcomes of HIV status disclosure among the HIV infected individuals. However, most of the HIV infected respondents confirmed referral to medical care and support groups, similar to another study of HBHCT in western Uganda [30]. Even with the linkage to care challenges, HBHCT can still play a major role since several studies demonstrate that knowing one’s status if a person is HIV-infected, has a substantial benefit in terms of risk reduction [41,42].

Several studies show that HBHCT increases uptake of HIV testing, identifies infected individuals earlier, and reaches more couples and children, in comparison to other HCT approaches [21,30]. Despite the ethical concerns, our study shows good adherence to standard HIV counseling and testing recommendations (consent, counseling, confidentiality and referral to care) and appreciation of the HBHCT approach by the community.

## Conclusion

This study also shows high coverage of HIV testing within the Kumi district community after HBHCT, with many individuals testing as couples. HBHCT can play a significant role in rapidly increasing access to HIV testing, care and treatment as well as prevention services.

## Competing interests

The authors declare that they have no competing interests.

## Authors' contributions

DK initiated the topic and wrote the first draft of the paper. RW and AH contributed to the design of the topic, interpretation of findings and writing of the paper. JK contributed to the interpretation and writing of the paper. All authors read and approved the final manuscript.

## Pre-publication history

The pre-publication history for this paper can be accessed here:

http://www.biomedcentral.com/1471-2458/12/966/prepub
